# Internet-Delivered Cognitive Behavior Therapy as a Prequel to Face-To-Face Therapy for Depression and Anxiety: A Naturalistic Observation

**DOI:** 10.3389/fpsyt.2019.00902

**Published:** 2020-01-09

**Authors:** Daniel Duffy, Angel Enrique, Sarah Connell, Conor Connolly, Derek Richards

**Affiliations:** ^1^ Clinical Research and Innovation, SilverCloud Health, Dublin, Ireland; ^2^ E-Mental Health Research Group, School of Psychology, Trinity College, Dublin, Ireland

**Keywords:** depression, anxiety, internet-delivered interventions, internet-delivered cognitive behavioral therapy (iCBT), Improving Access to Psychological Therapies (IAPT)

## Abstract

**Background:** The UK’s Improving Access to Psychological Therapies (IAPT) program is a stepped-care model treating individuals with depression and anxiety disorders. Internet-delivered cognitive behavioral therapy (iCBT) is routinely offered to individuals with mild to moderate symptoms, but its applicability to individuals with severe clinical symptoms and requiring a high-intensity intervention is relatively unknown. The current study sought to investigate the potential impacts of using iCBT as a prequel for patients requiring high-intensity treatment (HIT; face-to-face) for depression and anxiety in IAPT.

**Methods:** The study utilized an open study design. One hundred and twenty-four participants who were on a waiting list for high-intensity, face-to-face psychological treatment were offered iCBT. Psychometric data on symptoms of depression, anxiety, and functioning were collected from participants before starting and on finishing iCBT and at the point of service exit. Therapeutic alliance data were collected from patients and clinicians during treatment. Patient pathway data, such as number of treatment sessions and time in treatment, was also collected and incorporated into the analysis.

**Results:** Significant reductions across primary outcome measures of depression and anxiety, as well as improved functioning, were observed from baseline to iCBT treatment exit, and from iCBT exit to service exit. Analysis of the therapeutic alliance data for patients and clinicians illustrated differences in outcome for those who dropped out and those who completed treatment.

**Discussion:** This study illustrates the potential for using iCBT as a prequel to high-intensity therapy for depression and anxiety disorders and is the first of its kind to do so within IAPT stepped care. The results show that iCBT is a valuable option reducing waiting times and enhancing clinical efficiency. The study contributes to the well-established evidence on online psychological treatments worldwide, but further clinical and service development research is necessary to scale these treatments appropriately.

## Introduction

According to the Global Burden of Disease study, depression and anxiety disorders contribute the greatest degree of disability amongst all mental and substance abuse disorders (1). Psychological therapies have been shown to be both clinically and cost effective in the treatment and management of depression and anxiety disorders (2) and preferred by some over pharmacological interventions (3). In recent years, technology has facilitated the dissemination of psychological therapy, particularly cognitive behavior therapy, through internet-delivered interventions for depression and anxiety (4). Based on the available evidence for the efficacy of internet-delivered cognitive behavioral therapy (iCBT) for depression and anxiety across multiple meta-analyses (5–7), these interventions are currently deployed in a supported manner as part of routine care in mental health clinics in several countries (8). The literature shows that clients value the helpfulness of supporters, as they encourage and motivate clients to keep using the intervention as well as provide helpful guidance and feedback, which contribute to enhanced outcomes (9, 10, 11) Furthermore, iCBT is included as a treatment option in the UK clinical guidelines for the treatment of depression and anxiety disorders (12, 13).

Different authors advocate for the inclusion of iCBT interventions within stepped mental healthcare models (14, 15). Stepped-care models seek to match treatment intensity to client needs by providing the least intrusive and most effective intervention for the client upon entering services (16). The Improving Access to Psychological Therapies (IAPT) program is a stepped-care approach to psychological care for people with depression and anxiety within the National Health Service (NHS) in the UK (17). Specifically, the IAPT model delivers low-intensity psychological interventions (i.e. iCBT, guided self-help) (18) at step 2 (mild to moderate presentations of depression and anxiety disorders) and delivers high- intensity psychological interventions at step 3 (severe presentations of depression and anxiety disorders).

In the IAPT model, supporters are primarily psychological wellbeing practitioners (PWPs), graduate workers who are trained to provide low-intensity mental health services in primary care (18). PWPs are trained to identify and assess common presentations of mental health difficulties and work collaboratively with their clients to develop a treatment plan that best suits their needs (19) PWPs, as they encourage and motivate clients to keep using the intervention as well as provide helpful guidance and feedback, which contribute to enhanced outcomes (15–17). The work of the PWP is principally guided by the Reach Out Curriculum and advocates competencies in six core areas, namely, information gathering, information giving, shared decision-making, low-intensity treatment interventions, supervision and values, culture, diversity, and policy (19).

The deployment of iCBT as part of both routine care in mental health clinics and step 2 of IAPT is similar in that these interventions are offered to individuals with mild to moderate symptoms of depression and anxiety and generally exclude those with more severe presentations. Thus, most studies testing the efficacy of iCBT have focused on individuals with mild to moderate symptoms of depression and anxiety, leading to a less established evidence base for the use of iCBT with more severe presentations (20). Despite this, studies that have explored the effects of iCBT on those with severe presentations have illustrated positive effects (21, 22), even showing maintenance of improvements at follow-up (22, 23). A recent meta-analysis of studies of iCBT robustly demonstrates the relevance of iCBT for severe depression presentations; the authors argue that their findings should lay to rest the notion that iCBT should be limited to clients with milder depressions (7). This raises the possibility of using iCBT as a frontline intervention alongside high-intensity therapy (HIT) service provision for individuals with severe symptoms of depression and anxiety (22). In the IAPT stepped-care context, iCBT could be offered while service users wait for high-intensity treatment resources (face-to-face) to become available. To date, however, there are no data on the utility of iCBT as part of HIT service delivery in IAPT.

Therapeutic alliance refers to the collaborative nature of the interaction between therapist and client that emerges due to the affective bond between them, the agreement on the specific tasks in treatment, and the agreement on therapeutic goals (24, 25). Psychotherapy research findings indicate that alliance is related to therapeutic change (26–28) and overall satisfaction with care (29). In the field of internet interventions, research on therapeutic alliance with online supporters is scarce (30). A recent narrative review concluded that a positive therapeutic alliance can be formed in guided iCBT, with client ratings similar to those found in face-to-face treatment (31). This review also suggests that while an alliance may be formed online, the bond may be less important for the alliance to develop through this medium than in face-to-face therapy. Another review concluded that the therapeutic alliance in iCBT, as traditionally measured, has shown mixed results (32). There is opportunity to explore further the nature of the therapeutic alliance online and its relative contribution to outcomes. Given the established importance of the relationship between client and supporter in alliance and outcomes, it could be valuable to consider both client and clinician ratings to provide a comprehensive picture and inform the development and accurate deployment of iCBT as part of stepped care (30, 33–35). Also, some authors suggest that there is a need for studies that measure therapeutic alliance ratings at different points of treatment, as this could provide important information about alliance development (36).

The current study sought to examine clinical outcomes and the alliance online in delivering the SilverCloud iCBT for depression and anxiety disorders as a prequel to face-to-face therapy for clients with severe symptoms of depression and anxiety. Based on previous research of the SilverCloud platform and programs (37–39), the following research questions were developed:

1. Can clinical symptoms of severe depression/anxiety improve pre–post iCBT?
2. Do patients with severe depression/anxiety who complete iCBT continue to improve with subsequent face-to-face therapy?
3. Do patients with severe depression/anxiety develop a therapeutic alliance with the practitioner who supports them during iCBT, and is this maintained over time?
4. What do clinicians think about iCBT as a prequel to face-to-face therapy for patients with severe depression/anxiety?

## Materials and Methods

### Design

The study followed an uncontrolled feasibility design, On the one hand, it aimed at examining quantitatively clinical outcomes on depression and anxiety, functional outcomes in terms of work and social functioning, waiting time reduction, and therapeutic alliance between client and clinician in regards to step 3 services. Qualitatively, clinicians’ experiences about the acceptability of the online intervention as a prequel to high-intensity therapy at step 3 of IAPT were assessed.

### Setting

Recruitment took place within one IAPT mental healthcare service of a National Health Service Trust in England over a 9-month period, between September 2016 and June 2017. Like most other IAPT providers, the service experiences high levels of demand on their service and, consequently, they struggle with waiting lists due to a shortage of trained professionals who can provide HIT. HIT service provision at the site has undergone a transformation in its delivery of evidence-based treatments. The service introduced a therapeutic package for those requiring HIT. Service users were offered iCBT before commencing face-to-face treatment. Clients were monitored throughout the intervention, and any deterioration in their symptoms was responded to. Alternatively, if an appointment for face-to-face therapy (high-intensity therapy) became available, they were offered this treatment to begin with. Options available at step 3 for escalation include individual CBT (face-to-face and some online counseling), face-to-face delivered primary care counseling, or interpersonal therapy.

### Participants

Our participants included both clinicians who worked at the IAPT service and clients who were referred to the service and for whom HIT was indicated as suitable. Clients who were deemed suitable for a typical HIT intervention and subsequently selected iCBT as part of their therapeutic package were invited to the study by their clinician. Criteria for receiving HIT in IAPT consists of clients with more severe presentations of anxiety and/or depression, client presentations that did not subside with a low intensity (step 2) treatment, or having a disorder that is not typically seen at the low intensity level (e.g. social anxiety). As with all IAPT services, substance abuse that is actively contributing to symptoms is an exclusion criterion, and these clients are referred on to specialist services for treatment.

All clinicians and PWPs supporting clients on SilverCloud as a prequel to HIT were eligible to participate in the study. Therefore, clinician participants consisted of a combination of clinical psychologists, counseling psychologists, and PWPs employed by the service.

### Procedure

#### Client Procedure

Eligible participants were informed of the study through their clinician and invited to take part during their assessment appointment. On sign-up, participants were presented with information sheets for the study, and were also invited to discuss their participation with their clinician. Those willing to participate were then required to digitally sign to give their informed consent for participation. Those who declined consent or decided to withdraw from the study upon commencing treatment were requested to contact their clinician at the healthcare service, who would re-assess the client and assign them to SilverCloud treatment-as-normal or another intervention before their face-to-face appointment became available. Once participants finished their course of SilverCloud treatment or the waiting period came to an end, they progressed to either group therapy, face-to-face counseling, face-to-face CBT, or CBT delivered by a clinician *via* the internet.

Throughout their use of services, participants were asked to complete the minimum data set, as per the national requirements regarding IAPT services. In addition, they were asked to complete the Scale to Assess the Therapeutic Relationship—Patient Version (STAR-P), a measure of therapeutic alliance from the client’s perspective, during the 8-week supported period of the SilverCloud intervention.

#### Clinician Procedure

Clinicians were presented with a notification on their user accounts of the iCBT intervention that alerted them to the opportunity to participate in the research study. After reading the information sheet, they gave their consent to participate through their digital signature. Clinicians using the SilverCloud dashboard were able to monitor their client’s progress throughout the 8-week supported period of the intervention, and they regularly gave them feedback and responded to the work they had completed. Following each of these iCBT review sessions, clinicians were invited to complete the Scale to Assess the Therapeutic Relationship—Clinician Version (STAR-C) through their user accounts on the platform, which assesses the therapeutic relationship from the clinician’s perspective. Clinicians were also invited to participate in a qualitative semi-structured interview pertaining to acceptability of iCBT as a prequel to HIT.

### Risk Management

At initial assessment and throughout treatment, clients were assessed for risk in line with routine clinical practice. The initial assessment for entry into services included questions of whether clients could maintain their safety while on the waiting list. Those who exceeded the cutoff score for risk in terms of self-harm on the screening questions were not eligible to participate in the study and were referred for additional support. Integrated risk measures in the SilverCloud platform allowed for the monitoring of any changes in risk for clients throughout the program. For example, if the client scored above 0 on the self-harm item of the PHQ-9, an alert would be sent through to their clinician, who could then escalate it appropriately within the established clinical governance structure. It is important to note that SilverCloud was not presented to clients as a program capable of providing crisis support, and this was further emphasized through informed consent, the client information sheet, and the user contract. Significant adverse events (SAEs) were handled in-service by the clinical team and were escalated appropriately.

### Completers vs. Dropout

Treatment dropout was defined using the IAPT Care Spell End Code (40) which collects the reason for service exit as determined by the clinician. In this study, clients were categorized into two categories: completers comprising the service exit reasons “completed scheduled treatment” and “referred to other service” and dropouts comprising “dropped out of treatment (unscheduled discontinuation).” Other service exit reasons were not observed in this study population. As 10 clients were still in treatment by the study end point, their dropout status was classed as missing data.

### Medication Status

Medication status was defined using the patient’s medication status at assessment as recorded in the patient management system. The options could be: “prescribed and taking,” “prescribed and not taking,” and “not prescribed.” Prescribed and not taking and not prescribed were combined within the same group (no medication), since both groups were not taking medication.

### Intervention

SilverCloud delivers CBT-based online interventions for anxiety disorders, depression, and also comorbid depression and anxiety. Each program is compliant with National Institute for Health and Care Excellence (NICE) guidelines for the use of CBT in treatment, and is composed of eight modules that follow evidence-based CBT principles. They include tools such as self-monitoring and thought recording, behavioral activation, cognitive restructuring, and challenging core beliefs; all of which are central to the learning goals of the program. Research to date on the SilverCloud interventions has yielded significant positive clinical outcomes (41, 42).

Within the IAPT program, SilverCloud is typically delivered as a stand-alone low-intensity (step 2) intervention, or as an adjunct to high-intensity therapy. Specifically, for this study, participants were signed up at point of assessment and received reviews every 10–14 days *via* the platform. Once a participant is registered and assigned to a program on the iCBT platform, they receive a message from their clinician at their first login. This message welcomes them to the program, highlights its numerous aspects, and encourages them in the use of the program. Every fortnight, a clinician logs on and review participants’ progress, leaving feedback for them and responding to the work they have completed.

#### Data Collection

The following data were collected (**Table 1**):

**Table 1:** Study measures and assessment times.

Measure	Assessment	Time of assessment
**Client measures**		
Patient Health Questionnaire (PHQ-9)	Depression symptoms	Baseline, internet-delivered cognitive behavioral therapy (iCBT) exit, and service exit
Generalized Anxiety Disorder (GAD-7)	Anxiety symptoms	Baseline, iCBT exit, and service exit
Work and Social Adjustment (WSAS)	Work and social functioning	Baseline, iCBT exit, and service exit
Scale to Assess the Therapeutic Relationship—Patient Version (STAR-P)	Therapeutic alliance	Throughout treatment, after each progress review with clinician in iCBT
**Clinician Measures**		
Scale to Assess the Therapeutic Relationship—Clinician Version (STAR-C)	Therapeutic Alliance	Throughout treatment, at each review of client in iCBT
**Semi-structured Interviews**	Therapeutic alliance	One month post-trial

iCBT exit, end of iCBT treatment; service exit, end of step 3 Improving Access to Psychological Therapies (IAPT) treatment.

#### Participant Self-Reported Outcomes

Routinely Collected Data (The IAPT Minimum Data Set). This is a battery of psychometric measures common to all IAPT services collected as part of treatment-as-usual in all services. This battery primarily consists of the Patient Health Questionnaire-9 (PHQ-9), the Generalized Anxiety Disorder-7 (GAD-7), and the Work and Social Adjustment (WSAS). These questionnaires are administered to participants in all interventions at specific points in the patient pathway, for example at assessment, during treatment, and at post-treatment.

Patient Health Questionnaire-9 (43, 44) is a self-report measure of depression that has been widely used in screening, in primary care, and for research. The PHQ-9 items reflect the diagnostic criteria for depression outlined by the *Diagnostic and Statistical Manual of Mental Disorders, Fourth Edition—Text Revision* (*DSM-IV-TR*) (45). Summary scores range from 0 to 27, where larger scores reflect a greater severity of depressive symptoms. Cutoff scores for the PHQ-9 include none: 0–4, mild: 5–9, moderate: 10–14, moderately severe: 15–19, and severe: 20–27. The PHQ-9 has been found to discriminate well between depressed and non-depressed individuals using the clinical cutoff of total score ≥10, with good sensitivity (88.0%), specificity (88.0%), and reliability (.89) (43, 44).

Generalized Anxiety Disorder-7 (46); GAD-7 comprises seven items measuring symptoms and severity of GAD based on the *DSM-IV* diagnostic criteria for GAD. Scores on the GAD-7 range from 0 to 21, where a higher score reflects greater severity of anxiety symptoms. The GAD-7 has good internal consistency (α = .92) and good convergent validity with other anxiety scales (46). Higher scores indicate greater severity of symptoms. Cutoff scores for the GAD-7 include minimal: 0–4, mild: 5–9, moderate: 10–14, and severe: 15–21. The GAD-7 has increasingly been used in large-scale studies as a generic measure of change in anxiety symptomatology, using a cutoff score of 8 (47–49).

Work and Social Adjustment Scale is a simple, reliable (α > .75), and valid measure of impaired functioning (50). The measure contains five self-report items that look at how the disorder impairs the client’s ability to function day to day on five dimensions: work, social life, home life, private life, and close relationships. A score of 0–9 on the measure indicates subclinical symptoms, 10–19 is indicative of significant functional impairment but lower clinical symptoms, and above 20 suggests both severe clinical symptoms and functional impairment (41).

Scale to Assess the Therapeutic Relationship—Patient Version (51) is the client version of the scale to assess the therapeutic relationship in community mental healthcare. It was developed to be used in adult patients with mental health problems in psychiatric care. It is composed of 12 items and three subscales. The first subscale, positive collaboration, explores the general quality of the therapeutic relationship between patient and clinician. The second subscale, positive clinician input, illustrates the extent to which the client perceives their clinician positively regarding them. The third subscale, non-supportive clinician input, illustrate clients’ perceived problems within the therapeutic relationship. The range of scores for the STAR-P is 0 to 48, with a higher score suggesting a better therapeutic relationship. The measure has shown excellent psychometric properties.

#### Access Data

Several types of treatment access data were collected as part of this research project. These data contained the following variables:

- Waiting time between initial client triage and access to iCBT (in days)
- Time spent by each client in iCBT (in days)
- Waiting time between initial client triage and access to HIT (in days)
- Time spent by each client in face-to-face HIT (in days)
- Number of iCBT reviews received by each client
- Number of HIT sessions attended by each client

### Clinician-Reported Outcomes

Scale to Assess the Therapeutic Relationship—Clinician Version (51) is the clinician version of the scale to assess therapeutic relationship in routine scale. It was developed to be used in adult patients with mental health problems in psychiatric care. Similar to the client counterpart (STAR-P), the measure is composed of 12 items and three subscales. Firstly, positive collaboration, which explores the general quality of the relationship and the overall degree to which the relationship works. Secondly, positive clinician input measures to what extent clinicians encourage, understand, and support the patient. Lastly, emotional difficulties assesses the clinician’s feeling that they cannot empathize with and are not accepted by the patient. The range of scores for the STAR is 0 to 48, with a higher score suggesting a better therapeutic relationship. The measure has shown excellent psychometric properties.

Semi-structured interview. The semi-structured interview schedule for clinicians was developed *ad hoc* for the present study. It aimed to explore clinicians’ perceptions regarding the content of the program, their experience offering the reviews, and the clinical utility of the intervention being offered during the waiting period and before accessing HIT.

### Ethics

This study was carried out in accordance with the recommendations of the United Kingdom’s Research Ethics Service (52) with written informed consent from all participants (see section 2.4.1, *Client Procedure*, for procedure). All participants gave written informed consent in accordance with the Declaration of Helsinki. The protocol was approved by Wales Rec 7 (reference number: 16/WA/0257).

For participants that opted out of the SilverCloud intervention or the research, it is important to note that they were made aware that their place on the waiting list for services would not be jeopardized by their non-participation. Furthermore, for those who chose to partake in the intervention, their choice did not prolong their stay on the waiting list or their access to HIT.

### Statistical Analyses

Estimates of caseness, reliable change, and recovery were calculated using IAPT recovery criteria. A client is at “caseness” if they score above the clinical caseness threshold on the PHQ-9 (≥ 10) or GAD-7 (≥ 8). A client is classed as recovered if they move from caseness at baseline to below the clinical caseness threshold post-treatment. Reliable improvement is defined as a decrease in either or both measures greater than or equal to the reliable change index (PHQ-9 RCI = 6, GAD-7 RCI = 4) with no parallel increase in score on either measure greater than or equal to the RCI. Clients that both recover and show a reliable improvement in their score(s) are said to be in reliable recovery.

Treatment effects on primary outcome measures from baseline to iCBT treatment exit to service exit were assessed using linear mixed models fit with restricted maximum likelihood (REML) in the R (53) package lme4 v1.1-13 (54). This analysis was conducted separately for each measure, and missing data were assumed to be missing at random (MAR). Dropout status, defined according to the IAPT service exit reason as detailed in *Materials and Methods*, was included as a factor in each model. All participants attended part or all of the scheduled iCBT intervention. However, many participants did not thereafter attend any high-intensity treatment. To investigate whether this factor was associated with more positive or negative outcomes, clients were classified according to whether they attended at least one high-intensity treatment appointment (High Intensity Yes) or no high-intensity treatment appointments (High Intensity No). Medication status was also included in the model, where participants who were prescribed and not taking and those who were not prescribed were combined within the same group (medstatus No), and the other group referred to people who were prescribed and taking medication (medstatus Yes). Model selection was carried out by first creating a full model including all fixed factors (time point, dropout status, medication status, high-intensity attendance, and all interactions between them) and then performing backward elimination of effects from this full model. Backward elimination and model evaluation were completed using the step function from R package lmerTest v3.0-0 (55), which determined the optimum model. Post-hoc comparisons of least-square mean predictions from the optimum models were carried out using the R package lsmeans v2.27 (56).

Progression of the therapeutic relationship throughout iCBT treatment was modeled separately from the client’s perspective (with STAR-P scores) and clinician’s perspective (with STAR-C scores). For each measure, scores were modeled using a linear mixed model with fixed factors of time (a continuous variable: number of days after iCBT treatment start date), dropout status, and the interaction between them and client as a random factor. Post-hoc comparisons of least-square mean predictions at the predicted treatment exit day were carried out using the R package lsmeans v2.27.

Lastly, due to the low response rate from clinicians for the qualitative interviews, a descriptive–interpretative qualitative approach was used (57). This method was used to identify clinicians’ viewpoints regarding acceptability of the iCBT intervention as a prequel to step 3 services. Acceptability was defined as the degree to which the intervention, from the clinician’s viewpoint, was acceptable in terms of content, relevancy, and ability to engage the user in a therapeutic relationship. For the analysis of the interviews, these were transcribed, and thereafter, meaning units were extracted from the responses of the clinicians. Meaning units are parts of data that even standing out of context provide a piece of meaning to the reader. These meaning units were clustered together to form categories around acceptability. A more detailed description of these categories, including quotes that mirror each of them, is depicted in **Table 2**.

**Table 2:** Definition of the categories used in the qualitative analyses of Clinician views on acceptability.

Categories	Definition	Quotes
Content	Content concerned both the quality and format of the iCBT intervention and the clinicians’ perceptions of its necessity and utility for the user.	*“The little stories are quite good and the kind of case studies, we liked that” (*1*)*
		*“key concepts and strategies which apply to most things” (*2*)*
Responsiveness	Responsiveness concerned the action of writing for and responding to a user, the purpose of carrying out a review and whether it was an accurate and comprehensive manner in which to respond to the user’s needs.	*“I think it was all tailored and I suppose I would, I am happy to be enthusiastic and do the extra work at the beginning and then if the person is not responding, then I tend to write less” (*1*)*
		*“I think encouragement and motivation is part of it … for them to know that there is somebody that they can ask questions to if they are feeling stuck” (*2*)*
Relationship	Relationship concerned the perceived strength of the bond between the supporting clinician and the user.	*“There were very few people I felt I had any kind of relationship with … there were people that I had on SilverCloud who I then had for individual therapy and it was essentially like meeting a stranger” (*1*)*
		*“I guess if you are writing things down, you are more measured about what you say, you have time to think about” (*2*)*
Purpose	This category concerned the clinicians’ opinions on the iCBT package being offered and used as part of the care pathways for the clinicians and their service.	*“The least it gives you is behaviours, challenging your thoughts, looking at your behaviour. It gives you all of that foundation and if that saves us two sessions within therapy across the board, that can be huge” (*1*)*
		*“SilverCloud filled the space for those people that can’t think of anything worse than sitting in a room with other people and seem much more happy doing the work on their own” (*2*)*

## Results

### Overview

**Figure 1:**
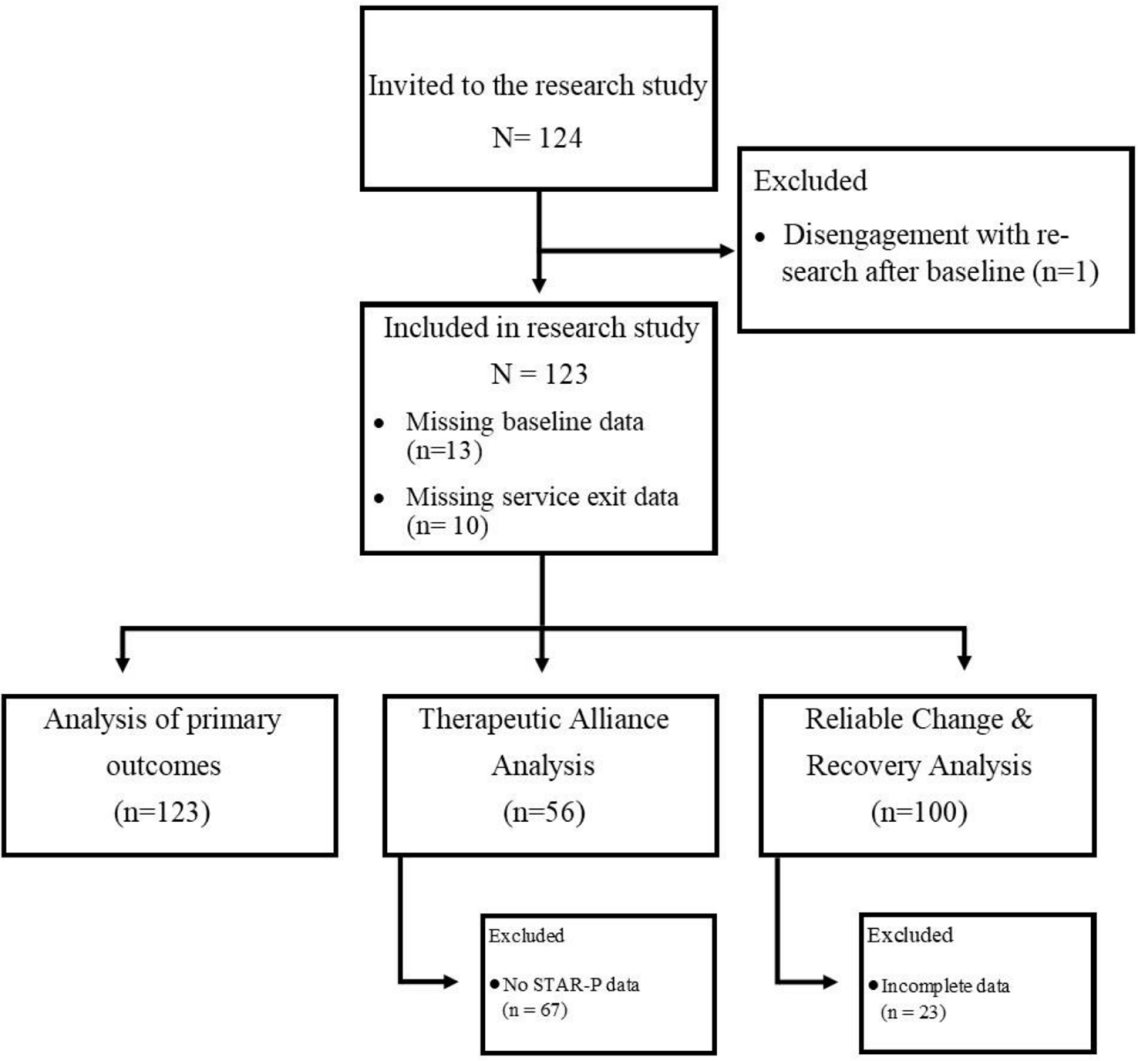
Trial flowchart, including numbers included in each statistical analysis.

One hundred and twenty-four (N = 124) clients were recruited to the study (**Figure 1**). One client did not engage after baseline, did not complete any post-assessment questionnaires, and therefore could not be included in analyses, leaving an effective sample size of N = 123. Sociodemographic characteristics of the population at baseline are presented in **Table 3**. Baseline scores were unavailable for 13 clients as the authors were unable to guarantee the integrity of their data at this time point due to a lack of clarity around previous treatment received and instances of a double assessment. Service exit scores were unavailable for 10 clients who were still in treatment by the end of the study. All 123 clients were included in analyses of primary outcome measures where models were fit with REML. However, the 23 clients with incomplete data could not be included in estimation of reliable change and recovery rates. All 123 clients were included in analyses of therapeutic alliance. Eleven clinicians were recruited and participated in the study.

**Table 3:** Sociodemographic characteristics of clients at baseline.

Variables	M (SD)	n (%)
**Gender**		
Female		85 (69)
Male		38 (31)
**Age**		
17–24		34 (28)
25–44		56 (46)
45–64		32 (26)
65–80		1 (1)
**Employment status**		
Employed		91 (74)
Homemaker/carer		6 (5)
Incapacity benefit		2 (2)
Retired		2 (2)
Student		13 (11)
Unemployed		8 (7)
Unknown		1 (1)
**Medication status**		
Not prescribed		55 (45)
Prescribed and taking		63 (51)
Prescribed but not taking		5 (4)
**Measures**		
PHQ-9 score	15.6 (5.5)	110 (89)
GAD-7 score	14.8 (4.5)	110 (89)
WSAS score	19.4 (8.7)	110 (89)
**Access Data**		
iCBT reviews	3.4 (2.0)	123 (100)
High-intensity treatment attended sessions	6.8 (6.4)	92 (75)
Time waiting for high-intensity treatment	158.7 ± 88.6 (days)	104 (85)
Time in iCBT treatment	46.8 ± 16.4 (days)	123 (100)
Time in high-intensity treatment	122.7 ± 99.0 (days)	92 (75)

### Treatment Adherence: Completers and Dropouts

One hundred and thirteen (*n* = 113) clients’ IAPT treatment episode had terminated by the study end point—68 completers and 45 dropouts as defined by their IAPT Care Spell End Code explained in the *Materials and Methods*. Ten clients were still in treatment by the study end point, so their dropout status was classed as missing data.

Completers attended on average 1.2 more iCBT appointments than dropouts [completers mean = 3.8, 95% CI (3.3, 4.3); dropouts mean = 2.6, 95% CI (2.1, 3.1); *t* (108) = 3.5, *p* < .001). There was no significant difference in the number of unattended iCBT appointments between completers and dropouts [completers mean = 1.9, 95% CI (1.5, 2.3); dropouts mean = 2.4, 95% CI (2.0, 2.7); *t* (109) = −1.9, *p* = .067]. There was no difference in the average amount of time in days spent in iCBT treatment [mean = 46.8, 95% CI (43.8, 49.7); *t* (91) = 1.3, *p* = .219].

Completers also attended on average 8.1 more high-intensity treatment appointments than dropouts [completers mean = 9.9, 95% CI (8.5, 11.2); dropouts mean = 1.8, 95% CI (0.6, 3.0); *t* (110) = 9.0, *p* < .001]. There was no significant difference in the number of unattended high-intensity treatment appointments between completers and dropouts [completers mean = 2.5, 95% CI (1.7, 3.2); dropouts mean = 2.1, 95% CI (1.4, 2.7); *t* (111) = 0.8, *p* = .437]. On average, completers spent almost twice the amount of days in high-intensity treatment than dropouts [completers mean = 147.2, 95% CI (122.9, 171.5); dropouts mean = 74.5, 95% CI (41.4, 107.6); *t* (63) = 3.6, *p* < .001].

### Reliable Change, Recovery, and Reliable Recovery

One hundred (n = 100) clients had a full set of questionnaire scores from all time points (baseline, iCBT exit, and service exit) and could be included in estimation of reliable improvement rates. Fifty-eight percent (58%; *n* = 58) exhibited reliable improvement from baseline to iCBT exit, and 70% (70 clients) exhibited reliable improvement from baseline to service exit.

Ninety-nine (n = 99) clients were above the clinical caseness threshold at baseline and could be included in estimation of recovery and reliable recovery rates. Recovery and reliable recovery rates for these 99 clients are detailed in **Table 4**. Twenty-two (*n* = 22) clients had achieved recovery by the time of iCBT exit and 20, of these had reliably recovered. Thirty-three (*n* = 33) clients were in recovery at the point of service exit, all of which had reliably recovered. The recovery rate was significantly higher for those 56 clients who completed treatment (46%) compared to the 43 clients who did not complete treatment (16%) (X-squared = 10.7, df = 2, *p* = .005).

**Table 4:** Recovery and reliable recovery rates for completers and dropouts.

Time points	N	Completers n (%)	Dropouts n (%)	χ^2^ (df)	*p*
Caseness at baseline	99	*56*	43		
**iCBT exit**					
Recovery	22	11 (20)	11 (26)	0.2 (1)	.645
Reliable recovery	20	9 (16%)	11 (26)	0.8 (1)	.360
**Service exit**					
Recovery	33	*26 (46)*	7 (16)	8.6 (1)	.003
Reliable recovery	33	*26 (*46*)*	7 (16)	8.6 (1)	.003

df, degrees of freedom.

### Primary Outcome Treatment Effects

For all three primary outcome measures, the optimum linear mixed model included only time point, dropout status, and the interaction of time point and dropout status as fixed effects and client as a random effect. High-intensity treatment attendance did not add significantly to any of the models indicating that dropout is a more accurate predictor of treatment outcome for all three measures. Medication status was found to be a significant variable in the model for PHQ-9 and not for GAD-7 and WSAS. Post-hoc comparisons within the PHQ-9 model did not find any interaction with any of the other variables and, therefore, was discarded for being included in further analyses (**Supplementary Table S1**).

Post-hoc analyses of the optimum linear mixed models showed a significant reduction of severity scores from baseline to iCBT exit and again from iCBT exit to service exit for all three primary outcome measures (**Table 5**). For all measures, a large reduction in severity score took place in the period from baseline to iCBT exit for both completers and dropouts. A further reduction was observed from iCBT exit to service exit only for completers. Dropouts showed no significant change.

**Table 5:** Differences in least-square mean questionnaire scores between time points.

Measure	Time point contrast	Least-squares mean (SE)	95% CI (lower–upper)	Least-squares mean (SE)	95% CI (lower–upper)	Estimated difference (SE)	95% CI (lower–upper)	*t* (df)	*p*
PHQ-9	Baseline–iCBT exit	15.408 (0.557)	14.31–16.51	11.837 (0.540)	10.77–12.91	*3.570* *(0.516)*	*2.55–4.59*	6.921 (220)	<.001
	iCBT exit–service exit: completers	11.529 (0.722)	10.1–12.96	7.456 (0.722)	6.03–8.89	4.074 (0.666)	2.76–5.39	6.116 (217)	<.001
	iCBT exit–service exit: Dropouts	12.145 (0.803)	10.56–13.73	12.298 (0.853)	10.61–13.99	−0.153 (0.795)	−1.73–1.42	−0.192 (222)	0.848
GAD-7	Baseline–iCBT exit	14.600 (0.478)	13.65–15.55	11.374 (0.465)	10.45–12.29	3.226 (0.430)	2.37–4.08	7.497 (221)	<.001
	iCBT exit–service exit: completer	10.912 (0.622)	9.68–12.14	6.926 (0.622)	5.69–8.16	3.985 (0.555)	2.89–5.08	7.177 (219)	<.001
	iCBT exit–service exit: Dropouts	11.836 (0.691)	10.47–13.20	12.001 (0.732)	10.55–13.45	−0.164 (0.663)	−1.48–1.15	−0.248 (224)	0.805
WSAS	Baseline–iCBT exit	19.151 (0.825)	17.52–20.78	16.725 (0.805)	15.13–18.32	2.426 (0.685)	1.07–3.78	3.542 (220)	0.001
	iCBT exit–service exit: completers	16.941 (1.076)	14.81–19.07	12.838 (1.076)	10.71–14.97	4.103 (0.883)	2.35–5.85	4.644 (218)	<.001
	iCBT exit–service exit: Dropouts	16.509 (1.197)	14.14–18.88	16.341 (1.258)	13.85–18.83	0.168 (1.056)	−1.92–2.26	0.159 (222)	0.874

iCBT, internet-delivered cognitive behavioral therapy; SE, standard error.

For the PHQ-9, scores were reduced by an average of 3.6 points from baseline to iCBT exit [Cohen’s d = 0.61, 95% CI (0.34, 0.88)], with no significant difference between completers and dropouts at either the baseline or iCBT exit time point. Completers had a further reduction of on average 4.0 points from iCBT exit to service exit [Cohen’s d = 0.64, 95% CI (0.29, 0.99)], whereas those who disengaged with the service showed no further change in score at service exit [Cohen’s d = −0.05, CI (−0.47, 0.37)].

Similarly, baseline GAD-7 scores were reduced by on average 3.2 points at iCBT exit time point [Cohen’s d = 0.69, 95% CI (0.42, 0.97)] and a further 4.0 points at service exit for completers [Cohen’s d = 0.83, 95% CI (0.47, 1.18)]. Dropouts did not show this further reduction in scores at service exit [Cohen’s d = 0.03, 95% CI (−0.39, 0.45)].

Change in WSAS score, which estimates severity of work and social adjustment impairment, followed a similar pattern with a substantial reduction (mean = 2.4 points) in severity score at iCBT exit time point for the whole population [Cohen’s d = 0.31, 95% CI (0.04, 0.58)]. Completers showed a further reduction of 4.1 points [Cohen’s d = 0.60, 95% CI (0.25, 0.94)], whereas dropouts did not have any significant change in score from iCBT exit to service exit [Cohen’s d = 0.15, 95% CI (−0.27, 0.57)].

### Therapeutic Alliance—Client’s Perspective

In total, 134 STAR-P questionnaires were collected, representing the therapeutic relationship of 56 clients from the client’s perspective. The total amount of time in days spent in iCBT treatment was similar for completers (mean = 47.279, SD = 15.757) and dropouts (mean = 46.145, SD = 17.274). No significant change in average STAR-P scores were seen for dropouts. For completers, a significant increase in STAR-P scores of on average 3.9 points was observed from baseline (day 0) to average end of treatment (day 46) (**Table 6**).

**Table 6:** Difference in least-square mean STAR-P total scores and sub-scores from baseline to iCBT treatment exit for completers and dropouts.

	Baseline	95% CI (lower–upper)	Treatment exit	95% CI (lower–upper)	Difference (SE)	95% CI (lower–upper)	t (df)	*p*
	Mean (SD)		Mean (SD)					
**STAR-P Total**								
Completers	34.100 (1.631)	33.81–34.39	37.410 (1.543)	37.13–37.69	−3.310 (1.036)	.5.36–1.26	−3.195 (82)	0.002
Dropouts	37.122 (2.104)	36.75–37.5	36.227 (2.129)	35.85–36.61	0.895 (1.306)	−1.69–3.48	0.685 (61)	0.495
**STAR-P PC**								
Completers	16.172 (1.175)	15.96–16.38	18.421 (1.114)	18.22–18.62	−2.249 (0.732)	−3.69, –.079	−3.071 (61)	0.003
Dropouts	18.300 (1.518)	18.03–18.57	16.934 (1.535)	16.66–17.21	1.365 (0.923)	−.046, –3.19	1.479 (62)	0.143
**STAR-P PCI**								
Completers	7.684 (0.572)	7.58–7.79	9.001 (0.532)	8.91–9.1	−1.317 (0.410)	−2.13, –.51	−3.21 (84)	0.002
Dropouts	8.622 (0.729)	8.49–7.75	8.564 (0.740)	8.43–8.69	0.058 (0.518)	−.097, –1.08	0.112 (82)	0.911
**STAR-P NCI**								
Completers	10.182 (0.434)	10.10–10.26	10.063 (0.368)	9.99–10.12	0.118 (0.430)	−.73, –.97	0.275 (95)	0.784
Dropouts	10.117 (0.528)	10.02–10.21	10.886 (0.534)	10.79–10.98	−0.769 (0.548)	−1.85, –.32	0.1.405	0.163

STARP PC, Positive Collaboration subscale of the STAR; STARP PCI, Positive Clinician Input subscale of the STAR; STARP NCI, Non-supportive Clinician Input subscale of the STAR.

### Therapeutic Alliance—Clinician’s Perspective

In total, 371 STAR-C questionnaires were collected, representing the therapeutic relationship of 82 clients from their clinician’s perspective. For completers, no significant change in STAR-C scores was observed from baseline (day 0) to end of treatment (day 47). For dropout clients, the STAR-C scores declined significantly by on average 5.4 points from baseline to end of treatment (day 46) (**Table 7**).

**Table 7:** Difference in STAR-C total scores and sub-scores from baseline to iCBT treatment exit for completers and dropouts.

	Baseline	95% CI (lower–upper)	Treatment exit	95% CI (lower–upper)	Difference (SE)	95% CI (lower–upper)	t (df)	*p*
	Mean (SD)		Mean (SD)					
**STAR-C Total**								
Completers	30.057 (1.542)	29.78–30.33	30.543 (1.500)	30.28–30.81	−0.486 (1.248)	−2.96–1.99	−0.389 (305)	0.697
Dropouts	31.695 (1.767)	31.38–32.01	26.281 (1.753)	25.97–26.59	5.414 (1.673)	2.10–8.73	3.236 (308)	0.001
**STAR-C PC**								
Completers	13.053 (0.808)	12.91–13.19	13.986 (0.783)	13.85–14.13	−0.933 (0.695)	−2.31–.44	−1.342 (309)	0.181
Dropouts	13.555 (0.929)	13.38–13.72	11.480 (0.922)	11.32–11.64	2.076 (0.932)	2.10–8.73	2.228 (311)	0.027
**STAR-C NCI**								
Completers	8.524 (0.417)	8.45–8.59	8.277 (0.408)	8.2–8.35	0.247 (0.319)	−.38–.88	0.776 (301)	0.438
Dropouts	9.054 (0.476)	8.97–9.14	7.411 (0.473)	7.33–7.49	1.643 (0.427)	.79–2.49	3.846 (304)	<.001
**STAR-C PCI**								
Completers	8.476 (0.411)	8.40–8.55	8.297 (0.400)	8.26–8.37	0.179 (0.332)	−.48–.84	0.54 (305)	0.59
Dropouts	9.088 (0.471)	9–9.17	7.395 (0.467)	7.31–7.48	1.693 (0.445)	.81–2.57	3.808 (308)	<.001

### Practitioner Feedback

Of the 11 clinicians, 2 consented to a follow-up interview to further explore their experiences about acceptability. One male CBT therapist and one female PWP constituted the interview sample. **Table 2** describes each of the four identified categories and supporting quotes from both participants.

## Discussion

The current study examined an innovative model of service delivery, integrating digital interventions as a frontline intervention before accessing HIT. On average, clients were on the moderate-severe range for depressive symptoms and on the severe range for anxiety symptoms at baseline. Regarding treatment outcomes, there was a decrease in symptoms upon completion of both iCBT and HIT interventions with effect sizes ranging from moderate to large, and around 20% of the sample achieved reliable recovery in advance of starting face-to-face therapy. These results corroborate previous results demonstrating the benefits of iCBT in reducing symptoms of depression and anxiety in more severe presentations of depression and anxiety (22). The results also indicate that iCBT can be beneficial as a prequel to high-intensity therapy in IAPT and in doing so facilitate clients to gain access to a frontline evidence-based intervention while waiting for face-to-face therapy, which is in line with the findings of other studies in blended interventions where internet interventions where offered before face-to-face therapy.

Despite the majority of clients transitioning from severe to mild presentations by point of service exit, some patients were still at caseness and, by definition, not recovered at this point. One potential explanation for these results is that, although current iCBT treatments are capable of producing improvements in symptoms, they may not be sufficient for producing lasting effects in severely anxious and depressed clients. Further treatments or booster sessions could therefore be necessary to achieve higher recovery rates (58, 59). This was especially noticeable in the dropout group, whose numbers achieved significantly less recovery rates at point of service exit. Completers received an adequate amount of HIT sessions to alleviate their symptoms (60), whereas those who dropped out completed only two HIT sessions on average and therefore any improvements made during iCBT were not further enhanced due to dropout from HIT. Understanding exactly what works for whom when integrating digital interventions into care pathways is an area of growing knowledge that would help to understand, and predict, future dropouts (61).

Clients spent on average 47 days in iCBT treatment and had 3.4 reviews on average during this period, meaning one review every 10 to 12 days. The observed administration of the intervention was below the intended use, that is, to be administered over a period of 8 weeks and for clients to receive six supporter reviews during this time (22, 62). This finding illustrates the importance of investigating the implementation of iCBT in novel contexts, since the ideal standard of intervention delivery was not adhered to in this study, but still produced clinical benefits. Further research regarding implementation of these interventions can illustrate and account for the changes that occur when interventions are implemented in novel contexts, as well as the barriers to implementation that are encountered (63). The value of the iCBT intervention is further reflected in the long waiting times clients experienced to accessing treatment. Average wait time was 158 days, and implementing iCBT can provide clients in these pathways with a frontline intervention in contexts where resources are scarce. In this instance specifically, waiting times for clients were reduced by 30%.

The dropout rate observed in this study (37%) is lower than the 45% rate of individuals not ending their course of therapy reported by IAPT (64). Reasons for dropout were not collected, but one study in this context found that waiting for an excessive period and the lack of contact during the wait lead people to dropout from services (65). In the current study, the long waiting list could have influenced people to disengage from services, and it might also have had an effect on improvement rates, as they have been shown to play a detrimental effect on outcomes (66). In regards to the wider literature on blended care interventions, a review conducted by Erbe et al. (67), found some studies where iCBT was also offered as a prequel to face-to-face therapy. The studies included in this review found dropout rates in the ranges of 30% to 59% across their samples (68–70), which is in line with what we observed in this study.

Therapeutic alliance from the client perspective increased significantly from pre to post for those who completed the scheduled intervention, while no change was observed in the case of those who dropped out before finishing their scheduled sessions. The positive alliance outcomes for completers showed that a strong therapeutic alliance can be established and maintained online (30, 31). These results further align with client perceptions of the role of support in iCBT, where different studies have shown that clients found it helpful and motivating during the intervention period (71, 72). The lack of change in perception of alliance for dropouts could be explained by the group receiving significantly less iCBT reviews and not getting enough contact to perceive an increase in alliance, which may take more time to develop in iCBT (73).

Therapeutic alliance from the clinician perspective showed the opposite pattern. Clinicians perceived no change in therapeutic alliance with clients who remained in treatment and perceived a significant decrease in alliance with those who dropped out from the services. The maintenance of alliance ratings perceived by the therapists over time on completers is similar to another study where these ratings were perceived as high, but no significant change was observed from pre- to post-treatment (34). The decrease in alliance with clients who dropped out could be explained by the fact that these clinicians conduct reviews for many clients in one scheduled review period and that they mostly base their perception of alliance on the usage of the platform while receiving little direct feedback from their client. In this sense, if they noticed some clients were engaging less with the platform, they may potentially feel a decrease in their perceptions of alliance over the course of the iCBT treatment. This interpretation is supported by the qualitative interviews, where clinicians said they put less effort in the reviews (i.e. write less and use more standard responses) if clients were not engaging, which could influence the levels of therapeutic alliance. Furthermore, these results could also be reflecting clinician bias toward relationship formation online, a result observed in other studies where clinicians also found iCBT as impersonal and thought that it was not feasible to create a therapeutic relationship in iCBT (74). However, more studies are needed to explore therapists’ perceptions of alliance in iCBT, and specially within regular clinical settings, in order to determine if these patterns are also observed in different settings and countries.

Although we gathered only a small amount of feedback from a small number of clinicians and therefore caution is advised in interpreting the comments, some interesting points arise. For starters, a positive aspect of the review process highlighted by clinicians was the asynchronous nature of the clinician–client contact, which allowed for them to reflect and provide more insightful feedback to their client. However, they reported that once they sent the message, there was no way for them to know how the client would interpret it. This uncertainty was further emphasized by the perceived removed nature of the therapeutic relationship, which they believed had a negative impact and made it difficult to establish a therapeutic alliance. Both clinicians agreed that the intervention would work best for clients who are self-motivated to work through the content with minimal guidance. These reports are in line with findings about the importance of the readiness to engage in therapy and the sense of self-directedness for iCBT (74), which have been found to be predictors of adherence (75). They also agreed that for step 3 service provision, an iCBT intervention should not be offered as a stand-alone solution but as an adjunct (prequel) to face-to-face therapy. This finding is also in line with the feedback obtained from therapists in other studies, who consider iCBT as an adjunct to face-to-face therapy more than a replacement and are more open to the possibility of offering blended interventions (74, 76, 77).

### Implications for Psychological Therapy Services

Some studies have explored the effects of internet and computerized CBT interventions as an adjunct to face-to-face therapy or as a treatment alternative in similar settings, showing mixed results. Thus, some studies have explored the beneficial effects of iCBT interventions when deployed at specialized care settings (78, 79), whereas others have not found any value in adding iCBT as an adjunct to face-to-face therapy (69). These inconsistencies in the findings may well be attributed to differences in implementation strategies and contextual factors, such as training with supporters, clinician’s attitudes toward the interventions, and level of integration of iCBT interventions into services and treatment pathways (80). Future studies should explore and consider the impact of different implementation factors on the uptake and effects of these interventions.

The findings observed in this study also have some implications for IAPT, where the national target for recovery is 50%, but there is no extant benchmark to untangle the recovery rates across different steps in IAPT. For the total sample, clients who received iCBT as a frontline intervention achieved a 22.2% recovery rate upon iCBT completion. Thereafter, with face-to-face therapy, this rate of recovery rose to 33.3%. Of importance, those who completed their entire course of treatment achieved a 46% recovery rate. These results provide initial supporting evidence for the use of iCBT as an adjunct to face-to-face therapies for more severe presentations. They do, however, stand in contrast to current guidance and practice in IAPT (12, 13) for the treatment of depression and anxiety, where digital interventions are recommended for clients with mild-moderate symptoms. As a consequence, these guidelines can also limit the scope and applicability of iCBT interventions to other contexts, such as high-intensity services for clients with more complex needs. Building on the work of prior research in the field (7, 21, 22) and the results from the current study, the authors acknowledge a need for further controlled research to robustly articulate the impacts of digital interventions in order to transform high-intensity service delivery. This will contribute to the development of clinical guidelines that incorporate digital interventions alongside current modalities of treatment for individuals with more severe presentations of depression and anxiety.

### Limitations

Naturalistic observation studies, such as this one, have some limitations due to their uncontrolled nature. In future studies, the magnitude of the effect produced by the intervention could be more clearly characterized through comparison with a control group with no access to the iCBT intervention. Primary outcomes were not collected before starting high-intensity therapy, and there was a gap between iCBT exit and the start of high-intensity therapy, so the true severity of symptoms when starting high-intensity therapy was unknown. Information about when the clients dropped out from services was unclear, making it difficult to establish whether effects could be attributed to iCBT or the HIT therapy. The current study recorded no SAEs for any participant, but it is important for the field of internet interventions to explore adverse events as they occur within natural service. For example, dropout from service is not rigorously followed up within IAPT, which limited the researchers in expanding further on those in the "dropout" group. In regards to the interviews with clinicians, the low response rate might have biased the feedback, where perhaps only those with a positive attitude toward digital interventions accepted to participate. The low response rate is likely to be due to the busy schedule the clinicians work under; however, future studies should confirm this hypothesis. Finally, date of referral was not collected, so we cannot ensure how long clients waited to access the iCBT program from referral. However, from IAPT reports and our experience with different services, the delay between referral and access to iCBT use to be minimal, and this was also probably the case in this study.

## Conclusion

The current research has illustrated the potential effectiveness and benefit of implementing an iCBT intervention as a prequel to face-to-face therapy in individuals with severe presentations of depression and anxiety in naturalistic settings. As indicated by Andersson and colleagues (81), open studies with no control group play a key role in clinical effectiveness research and, as such, the present paper constitutes a relevant contribution to the advancement of the field of iCBT. The results showed that iCBT was a valuable option regarding waiting time reduction and clinical efficiency. As stated by the clinicians participating in this research, communicating the foundations of CBT through an internet-delivered intervention can be of high value to a service regarding time, cost, and clinical efficiency. Although generalizability of the findings is limited by the uncontrolled nature of the research, future investigations into this area are warranted to further validate the potential benefits of incorporating iCBT as a frontline intervention to high-intensity services. The authors also acknowledge the need for research to be conducted on the implementation of these types of interventions into these contexts, using evidence-based methodologies, in order to determine how iCBT interventions can be best implemented into client pathways while accounting for levels of clinical risk. If implemented correctly within step 3 pathways, the clinical and cost benefits observed within mild-moderate clinical ranges and step 2 (low-intensity) services in IAPT could be successfully replicated and illustrated.

## Knowledge Contribution

Depression and anxiety disorders are a leading cause of disability worldwide. Internet-delivered cognitive behavior therapy (iCBT) has been successfully used to treat mild-moderate depression and anxiety disorders, but its applicability to more severe presentations is relatively undocumented. Typically severe presentations of depression and anxiety are treated using high-intensity interventions (HIT) such as face-to-face therapy. This study explored the potential benefits of iCBT as a prequel to face-to-face therapy for service users with depression/anxiety in a high-intensity therapy pathway for more severe presentations of depression and anxiety. Results from this preliminary research are promising; positive changes in clinical outcomes were observed for those completing iCBT, which further improved post-HIT. The relationship established between clinicians and patients was important and influenced by patient dropout. The results illustrate the potential utility of iCBT when used as a prequel to HIT. The research also presents a novel knowledge contribution to iCBT and the wider field of psychological therapies and paves the way for future investigations.

## Data Availability Statement

The datasets generated for this study are available on request to the corresponding author.

## Ethics Statement

This study was carried out in accordance with the recommendations of the United Kingdom’s Research Ethics Service with written informed consent from all subjects. All subjects gave written informed consent in accordance with the Declaration of Helsinki. The protocol was approved by Wales Rec 7 [reference number: 16/WA/0257]. For participants that opted out of the SilverCloud intervention or the research, it is important to note that they were made aware that their place on the waiting list for services would not be jeopardised by their non-participation. Furthermore, for those who chose to partake in the intervention, their choice did not prolong their stay on the waiting list or their access to step 3 interventions.

## Author Contributions

DD and DR conceptualized the initial design of the study. AE led on the development of the first and subsequent drafts of the manuscript, with significant contributions from both DD and SC. DD, AE, and SC designed the data analytic plan, and SC implemented it. CC conducted and analyzed the qualitative interviews. DR reviewed the manuscript and provided feedback for each draft.

## Conflict of Interest

DD, AE, SC, CC, and DR are all employees of SilverCloud Health. DD, AE, and DR are also researchers with the E-Mental Health Research Group of Trinity College, Dublin, Ireland. This research was funded by SilverCloud, the commercial company marketing the iCBT system studied here.

As principal funder for this research, SilverCloud Health provided the necessary research resource to design the study, collect, and analyze the data and write the report. The researchers adhere to the ethical standards of research and reporting of the National Health Service (NHS) and Trinity College Dublin (https://www.tcd.ie/research/dean/ethics/).
