# Supplementary material for: Internet-Delivered Cognitive Behavior Therapy as a Prequel to Face-To-Face Therapy for Depression and Anxiety: A Naturalistic Observation
Source: Front Psychiatry. 2020 Jan 9;10:902. doi: 10.3389/fpsyt.2019.00902 (PMC6962244; doi:10.3389/fpsyt.2019.00902)
Supplement: Supplementary file 1 [file Table_1.docx]

**Supplementary Table S1.**

*Backward reduced ANOVA tables for model parameter selection*

| **PHQ-9** | | | | | | | |
| --- | --- | --- | --- | --- | --- | --- | --- |
| **Fixed effect** | Eliminated | Sum Sq | Mean Sq | NumDF | DenDF | F value | Pr(>F) |
| highintensity:medstatus | 1 | 0.050 | 0.052 | 1 | 120.9 | 0.004 | 0.952 |
| dropout:medstatus | 2 | 1.440 | 1.440 | 1 | 116.9 | 0.099 | 0.754 |
| timepoint: highintensity | 3 | 18.400 | 9.200 | 2 | 221.6 | 0.631 | 0.533 |
| highintensity:dropout | 4 | 43.050 | 43.046 | 1 | 127.8 | 2.956 | 0.088 |
| highintensity | 5 | 14.300 | 14.296 | 1 | 122.9 | 0.980 | 0.324 |
| timepoint:dropout | 0 | 426.160 | 213.082 | 2 | 219.8 | 14.606 | <0.001 |
| timepoint:medstatus | 0 | 153.130 | 76.564 | 2 | 219.6 | 5.248 | 0.01 |
|  |  |  |  |  |  |  |  |
| **Random effect** | Eliminated | npar | logLik | AIC | LRT | Df | Pr(>Chisq) |
| <none> |  | 12 | -1041.8 | 2107.6 |  |  |  |
| 1 \| individual | 0 | 11 | -1089.8 | 2201.5 | 95.944 | 1 | <.001 |
|  |  |  |  |  |  |  |  |
| **GAD-7** | | | | | | | |
| **Fixed effect** | Eliminated | Sum Sq | Mean Sq | NumDF | DenDF | F value | Pr(>F) |
| timepoint:medstatus | 1 | 5.138 | 2.569 | 2 | 218.8 | 0.245 | 0.783 |
| timepoint: highintensity | 2 | 22.179 | 11.089 | 2 | 224.2 | 1.064 | 0.347 |
| highintensity:medstatus | 3 | 12.694 | 12.694 | 1 | 121.8 | 1.214 | 0.273 |
| dropout:medstatus | 4 | 19.731 | 19.731 | 1 | 118.0 | 1.887 | 0.172 |
| medstatus | 5 | 16.507 | 16.507 | 1 | 118.5 | 1.577 | 0.212 |
| highintensity:dropout | 6 | 48.050 | 48.050 | 1 | 129.2 | 4.590 | 0.034 |
| highintensity | 7 | 0.254 | 0.254 | 1 | 124.5 | 0.024 | 0.877 |
| timepoint: dropout | 0 | 303.533 | 151.766 | 2 | 222.5 | 14.476 | <0.001 |
|  |  |  |  |  |  |  |  |
| **Random effect** | Eliminated | npar | logLik | AIC | LRT | Df | Pr(>Chisq) |
| <none> |  | 12 | -985.5 | 1995 |  |  |  |
| 1 \| individual | 0 | 11 | -1041.0 | 2104.1 | 111.1 | 1 | <.001 |
|  |  |  |  |  |  |  |  |
| **WSAS** | | | | | | | |
| **Fixed effect** | Eliminated | Sum Sq | Mean Sq | NumDF | DenDF | F value | Pr(>F) |
| dropout:medstatus | 1 | 4.663 | 4.663 | 1 | 121.1 | 0.176 | 0.676 |
| timepoint: highintensity | 2 | 31.543 | 15.771 | 2 | 220.2 | 0.595 | 0.552 |
| timepoint:medstatus | 3 | 72.406 | 36.203 | 2 | 219.4 | 1.369 | 0.257 |
| highintensity:medstatus | 4 | 50.815 | 50.815 | 1 | 116.1 | 1.918 | 0.169 |
| medstatus | 5 | 40.302 | 40.302 | 1 | 117.6 | 1.521 | 0.220 |
| highintensity:dropout | 6 | 67.796 | 67.796 | 1 | 126.1 | 2.557 | 0.112 |
| highintensity | 7 | 1.888 | 1.888 | 1 | 122.9 | 0.071 | 0.790 |
| timepoint: dropout | 0 | 300.298 | 150.149 | 2 | 221.1 | 5.659 | 0.004 |
|  |  |  |  |  |  |  |  |
| **Random effect** | Eliminated | npar | logLik | AIC | LRT | Df | Pr(>Chisq) |
| <none> |  | 12 | -1156 | 2335.9 |  |  |  |
| 1 \| individual | 0 | 11 | -1225.4 | 2472.9 | 138.93 | 1 | <.001 |
|  |  |  |  |  |  |  |  |
